# A verification phase adds little value to the determination of maximum oxygen uptake in well-trained adults

**DOI:** 10.1007/s00421-023-05388-w

**Published:** 2024-01-18

**Authors:** Fabienne Bruggisser, Jonathan Wagner, Max Niemeyer, Raphael Schoch, Fabian Schwendinger, Arno Schmidt-Trucksäss, Raphael Knaier

**Affiliations:** 1 Department of Sport, Exercise and Health, Faculty of Medicine, University of Basel, Basel, Switzerland; 2 Department Medicine, Training and Health, Institute of Sports Science and Motologie, Philipps-University Marburg, Marburg, Germany

**Keywords:** Cardiopulmonary exercise tests, Oxygen consumption, Verification test, ${{\dot{\text{V}}}}$O_2max_, ${{\dot{\text{V}}}}$O_2peak_

## Abstract

**Purpose:**

The objective was to investigate if performing a sub-peak or supra-peak verification phase following a ramp test provides additional value for determining 'true' maximum oxygen uptake (${{\dot{\text{V}}}}$O_2_).

**Methods:**

17 and 14 well-trained males and females, respectively, performed two ramp tests each followed by a verification phase. While the ramp tests were identical, the verification phase differed in power output, wherein the power output was either 95% or 105% of the peak power output from the ramp test. The recovery phase before the verification phase lasted until capillary blood lactate concentration was ≤ 4 mmol·L^−1^. If a ${{\dot{\text{V}}}}$O_2_ plateau occurred during ramp test, the following verification phase was considered to provide no added value. If no ${{\dot{\text{V}}}}$O_2_ plateau occurred and the highest ${{\dot{\text{V}}}}$O_2_ (${{\dot{\text{V}}}}$O_2peak_) during verification phase was < 97%, between 97 and 103%, or > 103% of ${{\dot{\text{V}}}}$O_2peak_ achieved during the ramp test, no value, potential value, and certain value were attributed to the verification phase, respectively.

**Results:**

Mean (standard deviation) ${{\dot{\text{V}}}}$O_2peak_ during both ramp tests was 64.5 (6.0) mL·kg^−1^·min^−1^ for males and 54.8 (6.2) mL·kg^−1^·min^−1^ for females. For the 95% verification phase, 20 tests showed either a ${{\dot{\text{V}}}}$O_2_ plateau during ramp test or a verification ${{\dot{\text{V}}}}$O_2peak_ < 97%, indicating no value, 11 showed potential value, and 0 certain value. For the 105% verification phase, the values were 26, 5, and 0 tests, respectively.

**Conclusion:**

In well-trained adults, a sub-peak verification phase might add little value in determining 'true' maximum ${{\dot{\text{V}}}}$O_2_, while a supra-peak verification phase adds no value.

## Introduction

The maximum oxygen uptake (${{\dot{\text{V}}}}$O_2max_) is generally considered the gold standard for assessing cardiorespiratory fitness (Hill and Lupton 1923), widely applied to evaluate the efficacy of exercise intervention programs and changes in physical fitness (Blair et al. 1995), and used to predict all-cause mortality (Blair et al. 1995; Laukkanen et al. 2016). ${{\dot{\text{V}}}}$O_2max_ represents the upper limit of the physiological oxygen transport and utilization system (Bassett 2002; Fletcher et al. 2013; Franklin 2007; Poole and Jones 2017). It is determined by cardiopulmonary exercise testing (CPET) (Bassett 2002). The primary criterion for determining the attainment of a 'true' ${{\dot{\text{V}}}}$O_2max_, and thus the highest physiologically achievable value during CPET, is the occurrence of a ${{\dot{\text{V}}}}$O_2_ plateau in the severe intensity domain (Howley et al. 1995; Niemeyer et al. 2021; Poole and Jones 2017). However, even at maximum effort, a ${{\dot{\text{V}}}}$O_2_ plateau at the end of ramp-based CPET is identified in less than half of the participants (Knaier et al. 2019; Lucía et al. 2006; Wagner et al. 2020; Day et al. 2003). In the absence of a ${{\dot{\text{V}}}}$O_2_ plateau, secondary ${{\dot{\text{V}}}}$O_2max_ criteria including percentage of age-predicted maximum heart rate, respiratory exchange ratio, and rating of perceived exertion are commonly used to diagnose 'true' ${{\dot{\text{V}}}}$O_2max_ (Knaier et al. 2019; Midgley et al. 2007). However, the major criticism of the current ${{\dot{\text{V}}}}$O_2max_ criteria, including ${{\dot{\text{V}}}}$O_2_ plateau and secondary ${{\dot{\text{V}}}}$O_2max_ criteria, is that they are often sensitive to exercise test protocol, exercise type, and participant characteristics (Midgley et al. 2007). Too low or too high criteria thresholds lead to over- or underestimation, respectively, of participant's exhaustion, resulting in misclassification of 'true' ${{\dot{\text{V}}}}$O_2max_ (Knaier et al. 2019).

An alternative to ${{\dot{\text{V}}}}$O_2_ plateau and secondary ${{\dot{\text{V}}}}$O_2max_ criteria could be the so-called verification phase. This is an additive constant load exercise test to the limit of exercise tolerance performed directly after a brief recovery phase following a ramp-based CPET (Midgley et al. 2007; Poole and Jones 2017; Scharhag-Rosenberger et al. 2011; Rossiter et al. 2006). Concordance between the highest ${{\dot{\text{V}}}}$O_2_ (${{\dot{\text{V}}}}$O_2peak_) values achieved in the verification phase and ramp-based CPET (usually within 2–3% according to the ${{\dot{\text{V}}}}$O_2max_ test–retest reliability) provides evidence of achievement of 'true' ${{\dot{\text{V}}}}$O_2max_ during CPET (Costa et al. 2021; Dalleck et al. 2012). To date, several authors have reported such verification of 'true' ${{\dot{\text{V}}}}$O_2max_ (Costa et al. 2021). Accordingly, mean ${{\dot{\text{V}}}}$O_2peak_ in the verification phase did not differ from mean ${{\dot{\text{V}}}}$O_2peak_ determined in a ramp-based CPET (Costa et al. 2021). One argument for implementing a verification phase to determine ${{\dot{\text{V}}}}$O_2max_ is that a higher incidence of successfully verified ${{\dot{\text{V}}}}$O_2max_ compared with the incidence of ${{\dot{\text{V}}}}$O_2_ plateau during a ramp-based CPET has been documented (Midgley et al. 2006; Scharhag-Rosenberger et al. 2011). Although numerous studies have investigated the effectiveness of verification phase for determining ${{\dot{\text{V}}}}$O_2max_, there is currently no established recommendation for the ideal implementation of a verification phase and its clear added value. Thus, various verification phase protocols with different intensity and recovery phase duration have been applied so far (Costa et al. 2021).

Several studies have performed a verification phase with a supra-peak load, i.e., intensities above the peak power output (PPO) achieved during ramp-based CPET. The rationale underlying this is that during a constant exercise test above PPO, ${{\dot{\text{V}}}}$O_2_ increases to ${{\dot{\text{V}}}}$O_2max_ and thus provides a second ${{\dot{\text{V}}}}$O_2max_ value that can be compared to the previous one (Hill and Ferguson 1999; Hill and Smith 1999; Poole et al. 1988, 1990). However, this is only possible if premature exercise intolerance does not intervene (Jones et al. 2011; Poole and Jones 2017). To be considered, however, for endurance-trained participants, work rate increase in ramp-based CPET is rather high to prevent exceeding optimal test duration (Midgley et al. 2008; Yoon et al. 2007). This can cause excessive peak intensity in the verification phase that cannot be sustained long enough for ${{\dot{\text{V}}}}$O_2_ kinetics to enable ${{\dot{\text{V}}}}$O_2max_ to be reached (Iannetta et al. 2020). For trained athletes with fast ${{\dot{\text{V}}}}$O_2_ kinetics, it requires approximately 2:00 min to achieve ${{\dot{\text{V}}}}$O_2max_ (Caputo and Denadai 2008). However, in most studies using supra-peak intensity, verification phase duration was less than 2:00 min (Niemeyer et al. 2021). For example, a recent published study by Wagner et al. (2021) implementing a verification phase in well-trained adults with a supra-peak intensity of 105% of the PPO revealed little added value of a verification phase for determining ${{\dot{\text{V}}}}$O_2max_. This little added value was likely due to the fact that the supra-peak load, based on a previously performed ramp-based CPET with a rather high increase in work rate, could not be sustained for a sufficient duration to allow ${{\dot{\text{V}}}}$O_2_ to increase to ${{\dot{\text{V}}}}$O_2max_ (Wagner et al. 2021). Hence, the question arises whether a supra-peak verification phase can have additional value in determining ${{\dot{\text{V}}}}$O_2max_. It might be beneficial to conduct a verification phase below PPO since the loading can be sustained for a longer duration due to the reduced intensity. Indeed, not the PPO but rather the critical power represents the threshold intensity beyond which ${{\dot{\text{V}}}}$O_2max_ can theoretically be evoked (Hill and Ferguson 1999; Hill and Smith 1999; Poole et al. 1988, 1990). Thus, verification phase with exercise intensities above the critical power but below the PPO might be beneficial for determining ${{\dot{\text{V}}}}$O_2max_, particularly if the work rate increase of the ramp-based CPET is rather high (Iannetta et al. 2020).

Besides the intensity of the verification phase, the duration of the recovery phase between the ramp-based CPET and verification phase should be considered. Previous investigations found no significant effect of recovery phase duration on the difference between ${{\dot{\text{V}}}}$O_2peak_ achieved in a ramp test and verification phase (Costa et al. 2021). Regardless of the recovery phase lasting less than 15 min (Foster et al. 2007; Rossiter et al. 2006; Scharhag-Rosenberger et al. 2011; Sedgeman et al. 2013) or 24 h (Scharhag-Rosenberger et al. 2011), an identical mean ${{\dot{\text{V}}}}$O_2peak_ was obtained in the ramp test and verification phase. However, it is already known that prior vigorous or severe exercise increasing baseline blood lactate concentration to approximately 3–5 mmol·L^−1^, improves subsequent high-intensity cycling performance in well-trained adults (Burnley et al. 2005). Additionally, prior vigorous exercise has been shown to effectively accelerate the ${{\dot{\text{V}}}}$O_2_ response to exercise (Wilkerson et al. 2004) and increase the time to the limit of exercise tolerance during subsequent supra-peak exercise (Jones et al. 2003). Therefore, the question arises whether an optimal determination of the recovery phase duration, adapted to the individual blood lactate concentration of each participant, can result in an increased added value of a verification phase. To the author's knowledge, this has not been investigated in any study to date (Costa et al. 2021).

In previous analyses, ${{\dot{\text{V}}}}$O_2peak_ attained during CPET has mostly been compared to verification ${{\dot{\text{V}}}}$O_2peak_ only on group level (Costa et al. 2021), and the proportion of participants in whom ${{\dot{\text{V}}}}$O_2max_ could actually be verified was only reported in a few studies (Bowen et al. 2012; Schaun et al. 2021; Wagner et al. 2021; Murias et al. 2018; Mier et al. 2012). In addition, most studies focused only on the incidence of 'successful' verification and failed to capture the actual added value of conducting such verification phases in consideration of previously achieved ${{\dot{\text{V}}}}$O_2_ plateaus or the attainment of secondary ${{\dot{\text{V}}}}$O_2max_ criteria (Costa et al. 2021; Schaun 2017; Niemeyer et al. 2021).

Thus, this study aimed to investigate the usefulness of a verification phase protocol in which the factors of intensity and recovery phase duration are implemented optimally and individually for determining ${{\dot{\text{V}}}}$O_2max_ in well-trained male and female adults. In addition, the study aimed to clarify the added value of this verification phase protocol in relation to the presence of a ${{\dot{\text{V}}}}$O_2_ plateau and achievement of secondary ${{\dot{\text{V}}}}$O_2max_ criteria during previous ramp-based CPET testing.

## Materials and methods

### Study design

This study was a cross-sectional single-center randomized study conducted at the Department of Sport, Exercise and Health at the University of Basel, Switzerland. The study was conducted between September 2020 and June 2021 under consistent conditions (air humidity, 40–55%; room temperature, 20–22 °C). All procedures were approved by the Ethics Committee of Northwestern and Central Switzerland (EKNZ-2019-01697). Written informed consent was obtained from all participants before the start of the study. Participants attended two study visits, with a recovery phase of at least 24 h in between, and over a period of eight to ten days. To ensure equal testing conditions for all participants, standardized procedures and instructions were used. On both days, participants performed CPET using a ramp protocol followed by a verification phase. While the ramp tests were identical on both days, the verification test differed in workload. One verification phase was with sub-peak load (i.e., 95% of PPO achieved during ramp test) and the other was with supra-peak load (i.e., 105% of PPO achieved during ramp test). The order of the verification phase tests was randomized.

### Participants

Eligibility criteria for the study were age between 18 and 39 years, body mass index ≤ 27 kg/m^2^, and high cardiorespiratory fitness. Exclusively participants with a ${{\dot{\text{V}}}}$O_2max_ score ≥ 95th percentile (i.e., ≥ 55 mL·kg^−1^·min^−1^ for males and ≥ 51 mL·kg^−1^·min^−1^ for females) according to normative data of the American College of Sports Medicine references were considered eligible (ACSM 2010). Exclusion criteria were cardiovascular diseases, febrile infections within the past 14 days, any type of diabetes mellitus, hypertension (systolic blood pressure > 160 mmHg; diastolic blood pressure > 100 mmHg), and participation in another clinical trial within the past four weeks. Participants were instructed to maintain a balanced diet and adequate water intake 72 h before the laboratory appointments, refrain from exercising and drinking alcohol 24 h before, abstain from caffeine four hours before, refrain from eating two hours before, and to go to bed early the night before the measurement.

### Acquisition of participant characteristics

To assess participants' pre-exercise risk stratification, a questionnaire was completed (Shephard 1988) and a resting 12-lead electrocardiogram was recorded before the first measurement. Participants answering 'yes' to any of the questions of the questionnaire and/or exhibiting abnormalities in the electrocardiogram were examined by a physician before participating in the study. Body height and weight were determined to the nearest 0.5 cm and 0.1 kg, respectively. Participants' body fat content and lean body mass were determined using a four-segment bioelectrical impedance analysis (Inbody 720; Inbody Co. Ltd., Seoul, South Korea) at both laboratory appointments before each CPET.

### Cardiopulmonary exercise testing

During both laboratory appointments, CPET using a ramp protocol to the limit of exercise tolerance followed by a verification phase were performed on a cycle ergometer (Sport Excalibur; Lode Medical Technology, Groningen, The Netherlands). Participants were allowed to choose their pedaling cadence as long as it was maintained above 60 rpm. Throughout the exercise test, participants were verbally encouraged to reach the limit of their exercise tolerance. After a 3-min warm-up at 50 W, the work rate increased linearly with 30 W per minute until exercise intolerance. The ramp test was followed by a recovery phase at 50 W. The recovery phase lasted until capillary blood lactate concentration was ≤ 4 mmol·L^−1^ ensuring sufficient recovery before the verification phase. Thus, the recovery phase duration differed among participants, but was limited to 30 min, regardless of whether the value was reached or not. The rationale for this cut-off value is that exercise tolerance might be increased during a subsequent exercise bout if the recovery phase duration is long enough to reduce blood lactate concentration below 3–5 mmol·L^−1^ (Bailey et al. 2009; Burnley et al. 2005). Subsequent to the recovery phase, one of the two verification phase tests was performed to confirm the 'true' ${{\dot{\text{V}}}}$O_2max_ of the ramp test. Throughout the entire exercise testing, gas exchange was continuously measured breath-by-breath (MetaMax 3B; Cortex Biophysik GmbH, Leipzig, Germany). Data were averaged across 10-s intervals for analysis. ${{\dot{\text{V}}}}$O_2peak_ was defined as the highest consecutive 30 s of ${{\dot{\text{V}}}}$O_2_ and maximal respiratory exchange ratio as the highest value during the exercise testing. Heart rate was continuously recorded using a 12-channel electrocardiograph (Custo med GmbH, Ottobrunn, Germany). Rating of perceived exertion was assessed using the Borg scale 6–20 (Borg 1982) at rest, after warm-up, every 3 min during the ramp test to the limit of exercise tolerance, every 3 min during the recovery phase, and at the end of the verification phase. Capillary blood lactate concentration was analyzed using 10 μL of blood drawn from the earlobe at rest, after warm-up, immediately after reaching the limit of exercise tolerance during the ramp test, every 3 min during the recovery phase, and at the end of the verification phase (SuperGL Ambulance, Hitado Diagnostic Systems, Moehnesee, Germany). The highest value measured was defined as maximum blood lactate concentration. Before each study visit, volume and two-point gas concentration calibration were performed on the respective metabolic cart.

A ${{\dot{\text{V}}}}$O_2_ plateau was defined as an increase in ${{\dot{\text{V}}}}$O_2_ < 50% during the final 50 W of the ramp test compared to the individual increase in the submaximal intensity domain (Niemeyer et al. 2020). For this purpose, we calculated the slope of the ${{\dot{\text{V}}}}$O_2_-work rate relationship of the final 50 W and of the submaximal intensity domain (from 80 W to PPO-60 W) using linear regression analyses. As previously described, this definition allows the diagnosis of a ${{\dot{\text{V}}}}$O_2_ plateau with a risk of false plateau diagnoses of less than 5% (Niemeyer et al. 2020). Further, if the verification ${{\dot{\text{V}}}}$O_2peak_ was ± 3% of the ${{\dot{\text{V}}}}$O_2peak_ achieved in the ramp test, ${{\dot{\text{V}}}}$O_2max_ verification was accepted (Costa et al. 2021; Nolan et al. 2014). Secondary ${{\dot{\text{V}}}}$O_2max_ criteria were also analyzed to verify ${{\dot{\text{V}}}}$O_2max_ in the ramp test. These were defined as maximal respiratory exchange ratio ≥ 1.13, maximal heart rate ≥ 93% of 208-(0.7·age), maximal heart rate ≥ 96% of 210-age, and rating of perceived exertion ≥ 19 (Knaier et al. 2019; Wagner et al. 2020).

### Data analysis

**Fig. 1:**
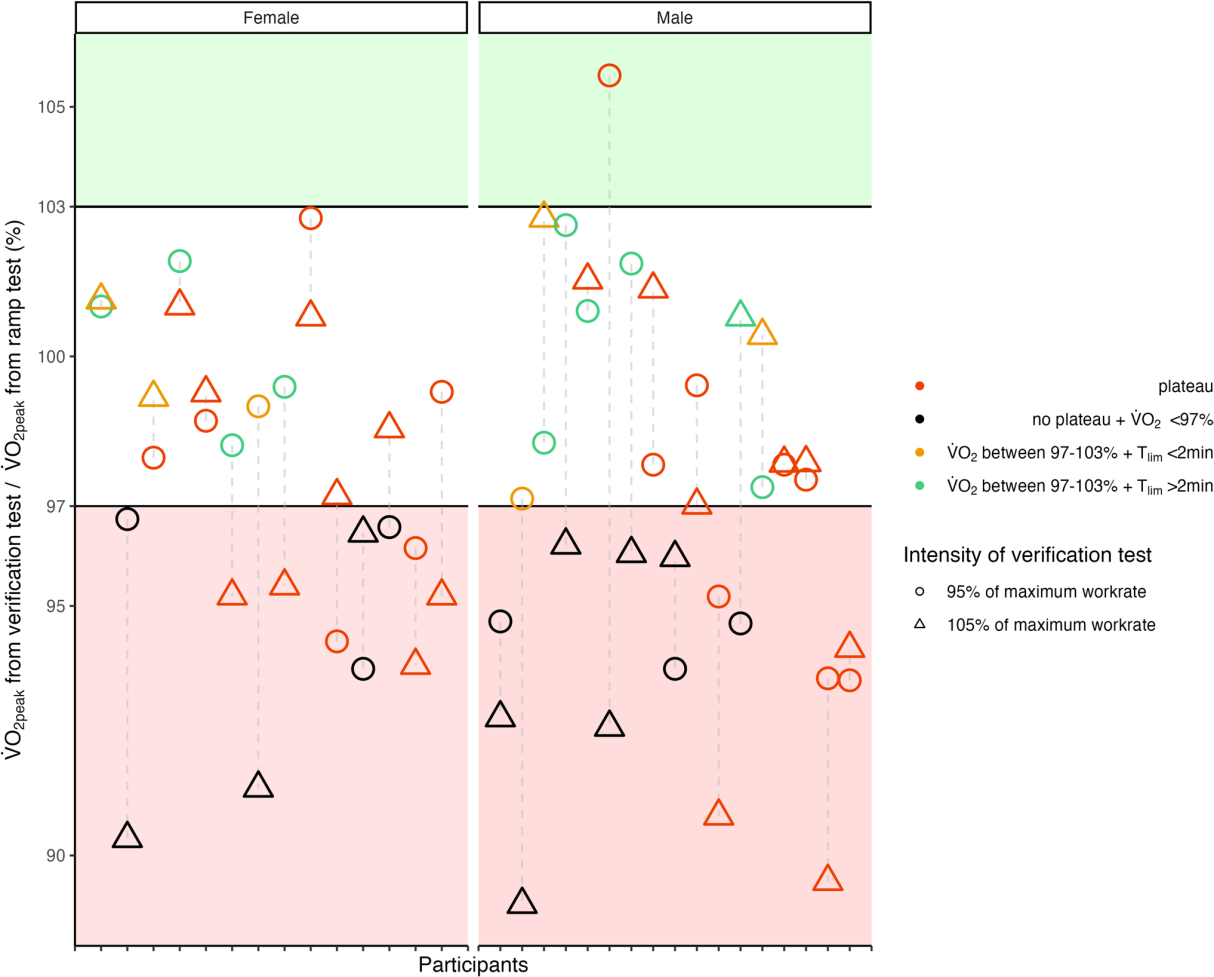
Ratio of verification ${{\dot{\text{V}}}}$O_2peak_ divided by ${{\dot{\text{V}}}}$O_2peak_ achieved in preliminary ramp test. Presented as a percentage for all tests conducted by participants. *T*_*lim*_ time to the limit of exercise tolerance, ${{\dot{\text{V}}}}$*O*_*2peak*_ highest oxygen uptake

**Fig. 2:**
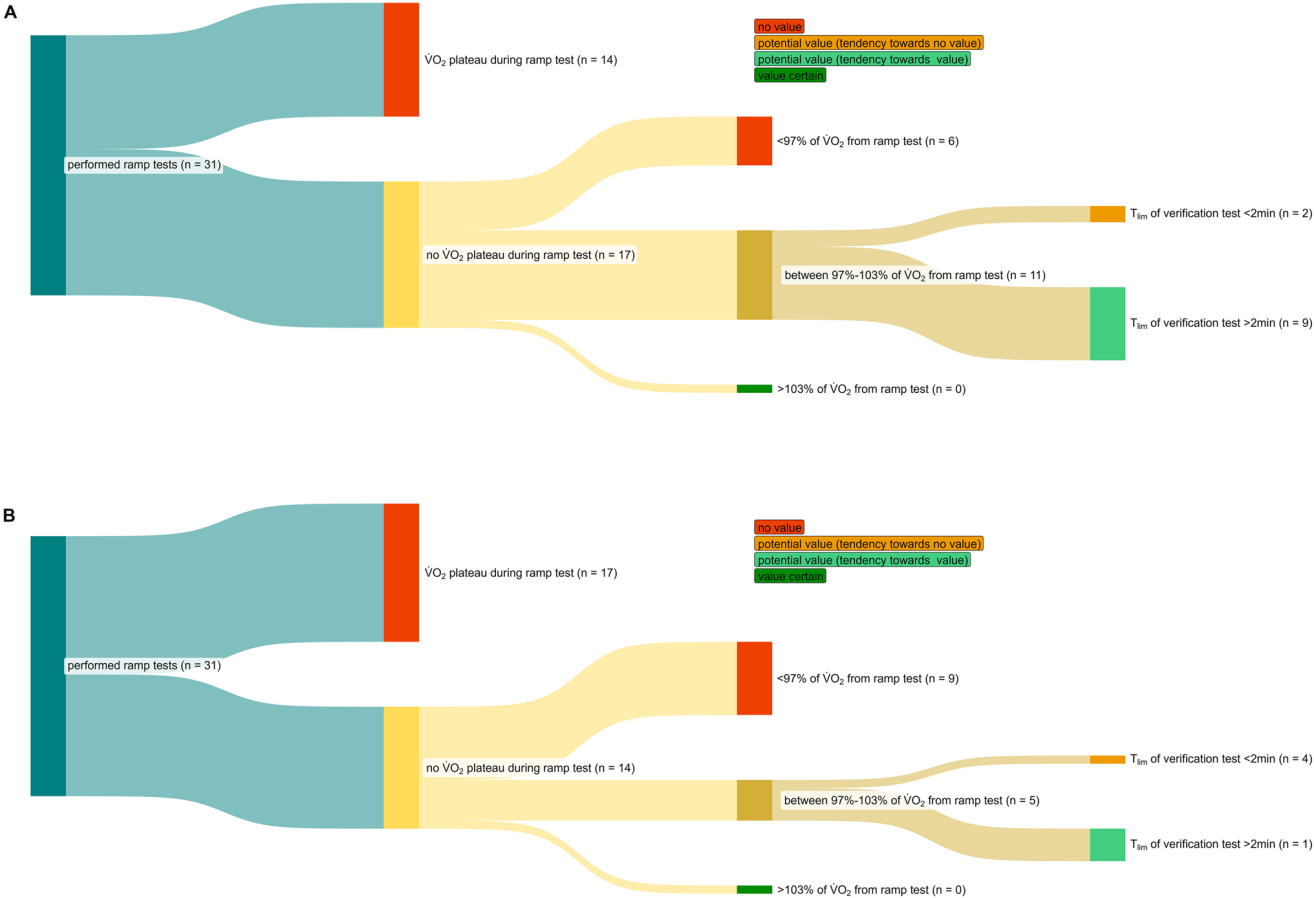
Classification of all 95% verification phases (**A**) and 105% verification phases (**B**) performed into no added value, potential added value, and certain added value

Data in text are presented as mean (standard deviation [SD]) unless noted otherwise. Descriptive statistics were applied to present participant characteristics, the results of both ramp tests, and the corresponding verification phase. A scatterplot was used to display the percentage of verification ${{\dot{\text{V}}}}$O_2peak_ (Fig. 1). The percentage was computed by dividing the verification ${{\dot{\text{V}}}}$O_2peak_ by the ${{\dot{\text{V}}}}$O_2peak_ achieved in the ramp test. To examine the additive value by performing a verification phase on the determination of the 'true' ${{\dot{\text{V}}}}$O_2max_, the percentage of tests was calculated for each of the subsequent three conditions: (1) no added value, (2) potential added value, and (3) certain added value (Fig. 2). No added value was defined if a ${{\dot{\text{V}}}}$O_2_ plateau occurred during the ramp test, indicating by itself that ${{\dot{\text{V}}}}$O_2max_ was achieved, or if no ${{\dot{\text{V}}}}$O_2_ plateau occurred and verification ${{\dot{\text{V}}}}$O_2peak_ was < 97% of the ${{\dot{\text{V}}}}$O_2peak_ achieved in the ramp tests. Potential added value was defined if no ${{\dot{\text{V}}}}$O_2_ plateau occurred and verification ${{\dot{\text{V}}}}$O_2peak_ was between 97 and 103% of the ${{\dot{\text{V}}}}$O_2peak_ achieved in the ramp test. This condition can be caused by two options: first ${{\dot{\text{V}}}}$O_2max_ was reached in the ramp test and confirmed by the verification ${{\dot{\text{V}}}}$O_2peak_; second ${{\dot{\text{V}}}}$O_2max_ was not reached in the ramp test but the time to the limit of exercise tolerance in the verification phase was too short to disprove a low ${{\dot{\text{V}}}}$O_2max_. Certain added value was defined if no ${{\dot{\text{V}}}}$O_2_ plateau occurred and verification ${{\dot{\text{V}}}}$O_2peak_ was > 103% of the ${{\dot{\text{V}}}}$O_2peak_ achieved in the ramp test, suggesting that the verification phase was effective in disproving a low ${{\dot{\text{V}}}}$O_2max_. The probability of verifying the occurrence of a ${{\dot{\text{V}}}}$O_2max_ during a ramp test with a verification phase depends on the time to reach the limit of exercise tolerance in the verification phase (Iannetta et al. 2020). Therefore, Pearson correlation between time to the limit of exercise tolerance in the verification phase and the difference between the ${{\dot{\text{V}}}}$O_2peak_ achieved in the ramp test and verification phase was calculated. Descriptive data are reported as mean and SD. A significance level of 0.05 was used for two-sided tests. For the analyses, IBM SPSS Statistics for Mac (Version 28, IBM, Armonk, NY, USA) was used. Figures were done in R version 4.1.2 (R-Core-Team 2021).

## Results

### Participants’ characteristics

Fifty-seven participants were assessed for eligibility. Seventeen participants did not meet the inclusion criteria for ${{\dot{\text{V}}}}$O_2max_, one participant was excluded due to the onset of the lockdown policies caused by the coronavirus disease 2019 pandemic, and one participant dropped out for personal reasons unrelated to the study participation. The remaining 38 participants completed the two laboratory appointments, whereby the values of seven of these participants had to be excluded due to technical measurement deficiencies (*n* = 3), face mask leakage (*n* = 2), and human error (*n* = 2). Finally, 17 males and 14 females were included in the analysis. Mean (SD) age, height, body mass, and body fat content were 23 (2) years, 176.8 (5.2) cm, 70.0 (5.2) kg, and 13.3 (5.2) %, for males (*n* = 17); and 22 (2) years, 165.9 (6.0) cm, 61.2 (8.2) kg, and 18.6 (6.2) %, for females (*n* = 14), respectively. Mean (SD) of ${{\dot{\text{V}}}}$O_2peak_ reached during the ramp test prior to the sub-peak verification phase was 64.5 (6.6) mL·kg^−1^·min^−1^ for males and 54.9 (5.9) mL·kg^−1^·min^−1^ for females. During the ramp test prior to the supra-peak verification phase, this value was 64.5 (5.9) mL·kg^−1^·min^−1^ for males and 54.8 (6.7) mL·kg^−1^·min^−1^ for females.

### Descriptive statistics

Descriptive findings from both ramp tests and the corresponding verification phase at 95% or 105% of the previously achieved PPO, respectively, are shown in Table 1. Twelve participants did not show a ${{\dot{\text{V}}}}$>O_2_ plateau in either ramp test, while in turn, twelve participants showed a ${{\dot{\text{V}}}}$O_2_ plateau in both ramp tests. In 15 (24.2%) of the 62 ramp tests, no ${{\dot{\text{V}}}}$O_2_ plateau was achieved and a verification ${{\dot{\text{V}}}}$O_2peak_ of less than 97% was reached (Fig. 2).

**Table 1:** Descriptive data for both ramp tests and the corresponding verification phase at 95% or 105% of the previously achieved peak power output, respectively

	RAMP before VER95			RAMP before VER105		
	Total	Males	Females	Total	Males	Females
	*n* = 31	*n* = 17	*n* = 14	*n* = 31	*n* = 17	*n* = 14
PPO (W)	363 (57)	403 (35)	315 (39)	362 (57)	401 (36)	314 (39)
T_lim_ (min)	11:33 (1:55)	12:52 (1:11)	9:57 (1:18)	11:30 (1:55)	12:49 (1:11)	9:55 (1:18)
${{\dot{\text{V}}}}$O_2peak_ (L·min^−1^)	3.98 (0.77)	4.51 (0.52)	3.35 (0.50)	3.98 (0.77)	4.50 (0.45)	3.33 (0.54)
HR_peak_ (bpm)	188.5 (8.4)	191.4 (8.9)	185.0 (6.6)	187.6 (8.5)	189.7 (8.9)	185.0 (7.4)
RER_peak_	1.21 (0.06)	1.20 (0.06)	1.22 (0.06)	1.20 (0.05)	1.20 (0.05)	1.19 (0.06)
RPE_peak_	19.8 (0.5)	19.8 (0.4)	19.7 (0.6)	19.9 (0.3)	19.9 (0.2)	19.8 (0.4)
∆${{\dot{\text{V}}}}$O_2_ < 50% (*n* [%])	14 [45.2]	8 [47.1]	6 [42.9]	17 [54.8]	8 [47.1]	9 [64.3]
HR_peak_ ≥ 96% of 210-age (*n* [%])	27 [87.1]	17 [100]	10 [71.4]	26 [83.9]	16 [94.1]	10 [71.4]
HR_peak_ ≥ 93% of 208-(0.7·age) (n [%])	29 [93.5]	17 [100]	12 [85.7]	27 [87.1]	16 [94.1]	11 [78.6]
RER_peak_ ≥ 1.13 (*n* [%])	26 [83.9]	14 [82.4]	12 [85.7]	26 [83.9]	15 [88.2]	11 [78.6]
RPE_peak_ ≥ 19 (*n* [%])	30 [96.8]	16 [94.1]	14 [100]	31 [100]	17 [100]	14 [100]

	VER95			VER105		
	Total	Males	Females	Total	Males	Females
	*n* = 31	*n* = 17	*n* = 14	*n* = 31	*n* = 17	*n* = 14
Recovery duration (min)	26:02 (3:40)	27:53 (2:46)	23:47 (3:25)	25:44 (3:22)	27:21 (2:59)	23:47 (2:45)
*T*_lim_ (min)	2:23 (0:24)	2:30 (0:25)	2:14 (0:20)	1:38 (0:16)	1:46 (0:14)	1:30 (0:13)
${{\dot{\text{V}}}}$O_2peak_ (L·min^−1^)	3.90 (0.75)	4.40 (0.50)	3.29 (0.50)	3.83 (0.72)	4.33 (0.43)	3.22 (0.52)
${{\dot{\text{V}}}}$O_2peak_ VER/RAMP (%)	98.0 (3.1)	97.7 (3.5)	98.3 (2.6)	96.5 (3.9)	96.3 (4.3)	96.8 (3.5)
${{\dot{\text{V}}}}$O_2peak_ VER/RAMP < 97% (*n* [%])	11 [35.5]	6 [35.3]	5 [35.7]	16 [51.6]	9 [52.9]	7 [50.0]
${{\dot{\text{V}}}}$O_2peak_ VER/RAMP 97–103% (n [%])	19 [61.3]	10 [58.8]	9 [64.3]	15 [48.4]	8 [47.1]	7 [50.0]
${{\dot{\text{V}}}}$O_2peak_ VER/RAMP > 103% (*n* [%])	1 [3.2]	1 [5.9]	0 [0]	0 [0]	0 [0]	0 [0]

Data are mean (standard deviation) or total numbers [percentages]

Note that ∆${{\dot{\text{V}}}}$O2 < 50% of the corresponding increase in ${{\dot{\text{V}}}}$O2 in the submaximal intensity domain indicates the occurrence of a ${{\dot{\text{V}}}}$O2 plateau, and that VER/RAMP < 97%, 97–103%, or > 103% represent no additional, potential, or clear added value of performing a VER, respectively, without considering the occurrence of a ${{\dot{\text{V}}}}$O2 plateau

*RAMP* ramp test, *VER95* verification phase at 95% of the previously achieved PPO, *VER105* verification phase at 105% of the previously achieved PPO, *PPO* peak power output, *T*_*lim*_ time to the limit of exercise tolerance, ${{\dot{\text{V}}}}$*O*_*2peak*_ highest oxygen uptake, ∆${{\dot{\text{V}}}}$*O*_*2*_ difference between the final and second-to-final 30 Watts, *RER*_*peak*_ highest respiratory exchange ratio, *HR*_*peak*_ highest heart rate, *RPE*_*peak*_ highest rate of perceived exertion

### Additional value of verification phase

Figure 1 shows the percentage of ${{\dot{\text{V}}}}$O_2peak_ achieved in each verification phase in relation to ${{\dot{\text{V}}}}$O_2peak_ achieved in the previous ramp test. Further, Fig. 2 presents the number of tests in which performing a verification phase at an intensity of 95% and 105% of the previously achieved PPO during ramp test, respectively, resulted in no added value, potential added value, and certain added value.

### Influence of recovery phase duration

Mean (SD) durations of the recovery phase before the 95% and 105% verification phase were 26:02 (3:40) minutes and 25:44 (3:22) minutes, respectively. All but one male participant had a blood lactate concentration of less than 4 mmol·L^−1^ by the end of the maximum 30-min recovery phase prior to both verification phases. Mean (SD) blood lactate concentration, heart rate, and rating of perceived exertion at the end of the recovery phase were 3.37 (0.55) mmol·L^−1^, 124 (13) bpm and 7.6 (1.9), immediately before the 95% verification phase; and 3.40 (0.49) mmol·L^−1^, 121 (12) bpm and 7.8 (1.9), immediately before the 105% verification phase, respectively.

### Influence of the time to the limit of exercise tolerance in verification phase

Mean (SD) time to the limit of exercise tolerance for the sub-peak and supra-peak verification phase were 2:23 (0:24) minutes and 1:38 (0:16) minutes, respectively. The Pearson correlation between the time to the limit of exercise tolerance in the sub-peak verification phase and the difference between the ${{\dot{\text{V}}}}$O_2peak_ achieved in the ramp test and verification phase was not significant (*r* = – 0.258; *p* > 0.161). Eleven of the 31 sub-peak verification tests resulted in potential value, whereas two of these eleven are unlikely to have added value because the duration of the verification phase was too short. In the remaining nine tests with potential value and a time to the limit of exercise tolerance longer than 2:00 min, almost all secondary ${{\dot{\text{V}}}}$O_2max_ criteria were reached during the previously performed ramp test. In detail, all nine participants reached a respiratory exchange ratio of ≥ 1.13 and a maximum heart rate ≥ 93% of 208-(0.7·age), whereas a maximum heart rate ≥ 96% of 210-age and a rating of perceived exertion ≥ 19 were both achieved by eight participants.

For the supra-peak workload, there was a significant negative Pearson correlation between the time to the limit of exercise tolerance in the verification phase and the difference between ${{\dot{\text{V}}}}$O_2peak_ achieved in the ramp test and verification ${{\dot{\text{V}}}}$O_2peak_ (*r* = – 0.417; *p* = 0.020). In only one out of five verification phases resulting in potential value, was the supra-peak power output maintained for > 2:00 min (Fig. 2b). Thus, the supra-peak verification phase might have added value in only one out of 31 participants. Of note, as already seen for the sub-peak test, this one participant reached all applied cutoffs for secondary ${{\dot{\text{V}}}}$O_2max_ criteria in the previously conducted ramp test.

## Discussion

The main finding of the present study is that sub-peak and supra-peak verification phases following a ramp test both add little value to determining ${{\dot{\text{V}}}}$O_2max_ in well-trained male and female adults. For the sub-peak test, ${{\dot{\text{V}}}}$O_2max_ could be confirmed with certain value in none and potential value in just 11 of 31 tests. Further, in only 9 of these 11 tests, the power output was sustained long enough to even enable reaching ${{\dot{\text{V}}}}$O_2max_. For the supra-peak test, ${{\dot{\text{V}}}}$O_2max_ could be confirmed with certain value in none and potential value in 5 tests, of which 4 showed an insufficient verification test duration of < 2:00 min. Half of all verification tests are obsolete because a ${{\dot{\text{V}}}}$O_2_ plateau is evident during the ramp test. In further 25% of participants, ${{\dot{\text{V}}}}$O_2_ is < 97% of the ramp test, and in further 10% of participants, the verification phase cannot be sustained long enough to enable verification of ${{\dot{\text{V}}}}$O_2max_. Hence the benefits of a verification phase seem minor in comparison to the higher burden for participants and staff.

### Additional value of a verification phase

In the absence of a ${{\dot{\text{V}}}}$O_2_ plateau, verification ${{\dot{\text{V}}}}$O_2peak_ at 95% and 105% intensity was less than 97% in 35.3% and 64.3% of the tests, respectively, implying that the verification phase also provided no additional benefit. Furthermore, verification ${{\dot{\text{V}}}}$O_2peak_ at 95% and 105% intensity was between 97 and 103% of ${{\dot{\text{V}}}}$O_2peak_ achieved in the ramp test in 64.7% and 35.7% tests, respectively. Here, the performed verification phase provided a potential added value. This uncertainty relies on two options: either ${{\dot{\text{V}}}}$O_2max_ was already reached in the ramp test and confirmed by verification ${{\dot{\text{V}}}}$O_2peak_, or ${{\dot{\text{V}}}}$O_2max_ was not reached in the ramp test but the time to the limit of exercise tolerance in the verification phase was too short to disprove a low ${{\dot{\text{V}}}}$O_2max_. Importantly, in none of the tests failing to reach a ${{\dot{\text{V}}}}$O_2_ plateau did the implementation of a verification phase provide certain added value. This applied to both verification phases at 95% and 105% of the PPO achieved in ramp test. Hence, it is evident that performing a verification phase does not yield any additional information on ${{\dot{\text{V}}}}$O_2max_ beyond what is already encompassed by the ${{\dot{\text{V}}}}$O_2_ plateau definition.

Considering the findings reported above, implementing our verification phase protocol with an optimal and individualized determination of the intensity and recovery phase duration does not seem to provide a clear additional value in determining ${{\dot{\text{V}}}}$O_2max_ in well-trained male and female adults. These findings support the results of a recently published meta-analysis by Costa et al. (2021), which examined apparently healthy adults aged between 19 and 68 years. Overall, the authors concluded that although the verification phase is a robust method for confirming the attainment of ${{\dot{\text{V}}}}$O_2max_ during a ramp test, the added value is questionable because the difference in ${{\dot{\text{V}}}}$O_2peak_ between ramp test and verification phase is only small (Costa et al. 2021). This agreement between the achieved ${{\dot{\text{V}}}}$O_2peak_ in the ramp test and verification phase was not influenced by ramp test protocol, recovery phase (type and duration), and verification phase protocol (intensity and duration) (Costa et al. 2021).

### Effects of the verification phase protocol and chosen analytical approach

In this study, a newly conducted verification phase protocol was applied. Most previous studies used only supra-peak verification phase intensity, recovery durations between 3 and 20 min, and group-level analyses (Costa et al. 2021). Moreover, these studies only examined whether verification phases are valuable in determining 'true' ${{\dot{\text{V}}}}$O_2max_ (Costa et al. 2021). However, a more expedient question may be, if verification phases provide added value beyond the use of a ${{\dot{\text{V}}}}$O_2_ plateau during the ramp test. The rationale for this is that the presence of a ${{\dot{\text{V}}}}$O_2_ plateau can be identified during a ramp test not necessitating additional burdensome examinations. Furthermore, a ${{\dot{\text{V}}}}$O_2_ plateau is the primary criterion to determine ${{\dot{\text{V}}}}$O_2max_ (Howley et al. 1995; Niemeyer et al. 2021; Poole and Jones 2017). In contrast, the present study investigated the usefulness of a sub-peak verification phase in addition to a supra-peak verification phase, implemented exhaustion-depended recovery duration, analyzed the collected data on an individual level, and considered the added value of a verification phase beyond a ${{\dot{\text{V}}}}$O_2_ plateau during ramp test. Rationales for choosing this verification phase protocol are as follows. First, considering that during supra-peak verification phase the limit of exercise tolerance might be reached prematurely, and thus the physical strain cannot be sustained long enough for the ${{\dot{\text{V}}}}$O_2_ kinetics to allow ${{\dot{\text{V}}}}$O_2max_ to be confirmed by a plateau (Caputo and Denadai 2008; Hill et al. 2002), a sub-peak verification phase was implemented additionally. Second, by monitoring the duration of the recovery phase before a verification phase through measuring blood lactate concentration, the effect of inadequate recovery can be controlled. Therefore, the occurrence of relevant disturbances in the skeletal muscle milieu due to metabolic acidosis can be excluded when analyzing the data (Schaun 2017). And third, most studies published to date have compared the ${{\dot{\text{V}}}}$O_2peak_ achieved during ramp test and verification phase averaged across study participants (Costa et al. 2021). Indeed, Costa et al. (2021) concluded a successful verification, since there were no significant group-level differences between the two ${{\dot{\text{V}}}}$O_2peak_ values. However, this is not sufficient for the individual athlete, as the results of the ramp test and the verification phase need to be compared at the level of the individual athlete, taking into account an existing ${{\dot{\text{V}}}}$O_2_ plateau, in order to determine whether the implementation of a verification phase can add value to determining 'true' ${{\dot{\text{V}}}}$O_2max_. To the authors' knowledge, although previous studies with healthy participants have performed individual analyses and assessed the achievement of a ${{\dot{\text{V}}}}$O_2_ plateau during the ramp test (Mier et al. 2012; Murias et al. 2018), the analytical approach described above to determine the added value of a verification phase has never been applied in this way, except in the study by Wagner et al. (2021).

### Usefulness of sub-peak verification phase

That ${{\dot{\text{V}}}}$O_2max_ achieved in a ramp test can be verified by a sub-peak verification phase has already been shown in several studies (Day et al. 2003; Niemeyer et al. 2019, 2020; Rossiter et al. 2006; Sedgeman et al. 2013). The meta-analysis by Costa et al. (2021) underlines this finding. Thus, differences between the ${{\dot{\text{V}}}}$O_2peak_ achieved in a ramp test and verification phase do not differ as a function of the chosen verification phase intensity (Costa et al. 2021). However, it should be noted that verifying ${{\dot{\text{V}}}}$O_2max_ using a sub-peak verification phase requires intensities above the critical power (Hill and Ferguson 1999; Hill and Smith 1999; Poole et al. 1988, 1990). Hence, determining the optimal intensity of the sub-peak power output is essential. To note, longer ramp test duration results in a PPO closer to critical power (Morton 2011; Sedgeman et al. 2013). Conceivably, intensities substantially lower than the PPO might cause verification intensity to be below critical power and, therefore, inhibiting attainment of ${{\dot{\text{V}}}}$O_2max_ (Sedgeman et al. 2013). That the sub-peak intensity used in the present study is above the critical power can be confirmed by two factors: first, the sub-peak verification phase yielded a ${{\dot{\text{V}}}}$O_2peak_ that was not different from the ${{\dot{\text{V}}}}$O_2peak_ achieved during the ramp test, and second, the time to the limit of exercise tolerance could only be sustained over a period of 2:23 min. Since ${{\dot{\text{V}}}}$O_2max_ can only be achieved above critical power during constant exercise testing (Hill and Ferguson 1999; Hill and Smith 1999; Poole et al. 1988, 1990) and intensities below critical power can be maintain for a very long time without fatigue occurring (Monod and Scherrer 1965), the aforementioned factors indicate that the intensity of 95% of the PPO must be above critical power and consequently suitable for testing ${{\dot{\text{V}}}}$O_2max_.

### Usefulness of a supra-peak verification phase

As mentioned beforehand, the time to the limit of exercise tolerance is the commonest limitation in the analysis and interpretation of verification test data. Several authors concluded ${{\dot{\text{V}}}}$O_2max_ is reached when the ${{\dot{\text{V}}}}$O_2peak_ in the ramp test and the verification phase only deviate by 2–3% (Costa et al. 2021; Dalleck et al. 2012). However, as Wagner et al. (2021) pointed out, this is just one approach. Another explanation might be the inability to sustain the verification power output long enough for the ${{\dot{\text{V}}}}$O_2_ kinetics to allow reaching ${{\dot{\text{V}}}}$O_2max_, and that the verification ${{\dot{\text{V}}}}$O_2_ would indeed be even higher. This approach has been illustrated in Fig. 1 in Wagner et al. (2021). The importance of the time to the limit of exercise tolerance in verification phases arises from findings showing that both healthy young individuals as well as runners and cyclists with fast ${{\dot{\text{V}}}}$O_2_ kinetics appear to require approximately 2:00 min to reach their ${{\dot{\text{V}}}}$O_2max_ (Caputo and Denadai 2008; Hill et al. 2002).

In our study, overall, only 3 out of 31 participants reached a sufficient duration of more than 2:00 min in the 105% verification phase. Notably, in 2 of these 3 participants, ${{\dot{\text{V}}}}$O_2max_ was already confirmed by a ${{\dot{\text{V}}}}$O_2_ plateau in the previous ramp test. The observed negative correlation between the duration of the supra-peak verification phase and the difference between the highest ${{\dot{\text{V}}}}$O_2_ achieved in the ramp test and verification phase may have resulted from the participants' inability to sustain the supra-peak loading long enough for ${{\dot{\text{V}}}}$O_2_ to reach 'true' ${{\dot{\text{V}}}}$O_2max_. The small number of participants able to maintain the duration for a sufficiently long period is consistent with most studies that have assessed the validity of supra-peak verification phases in healthy untrained, non-specific or specific trained adults (Niemeyer et al. 2021). When considering studies with either specific or non-specific trained participants, fast-increasing work rates in the ramp test, ranging from 25 to 30 W per minute, result in substantially higher PPO (Morton 2011). Consequently, the power output in the verification phase should be linked to the work rate increase in the ramp test. In many studies published to date, an insufficient duration of the supra-peak verification phase to reach ${{\dot{\text{V}}}}$O_2max_ was observed, regardless of the implemented intensity (between 105 and 110%) or type of exercise (running or cycling) (Astorino and DeRevere 2018; Nolan et al. 2014; Rossiter et al. 2006; Sedgeman et al. 2013). This is further emphasized in the meta-analysis from Costa et al. (2021). However, it is important to note, that just because in many previous supra-maximal verification phase protocols participants were not able to maintain the duration for a sufficiently long period, this does not mean that it is not possible. The development of “better” protocols could provide additional value in determination of ${{\dot{\text{V}}}}$O_2max_/${{\dot{\text{V}}}}$O_2peak_ in future. For example, Gaesser et al. (1995) claimed that the participants in their study were able to sustain a supra-maximal constant load for at least one minute in duration. However, they did not present the respective data. In participants with very fast ${{\dot{\text{V}}}}$O_2_-kinetics this duration could be sufficient to reach ${{\dot{\text{V}}}}$O_2max_.

### Comparison between sub-peak and supra-peak verification phases

In the present study, ${{\dot{\text{V}}}}$O_2max_ was more often verified by the verification phase at 95% than at 105% of PPO achieved in the ramp test (Fig. 2). While the 95% verification phase power output was maintained on average for 2:23 (0:24) minutes, the duration of the 105% verification phase averaged only 1:38 (0:16) minutes. Thus, the time to the limit of exercise tolerance in the 95% verification phase is clearly above the 2:00 min required for healthy young individuals as well as runners and cyclists to allow the ${{\dot{\text{V}}}}$O_2_ kinetic to reach 'true' ${{\dot{\text{V}}}}$O_2max_ (Caputo and Denadai 2008). Hence, for well-trained male and female adults, performing a sub-peak verification phase seems to be more beneficial than performing a supra-peak verification phase. Note, although sub-peak verification phases may appear preferable, they are still of limited value in determining 'true' ${{\dot{\text{V}}}}$O_2max_ in a cohort of well-trained participants, especially when considering the presence of a ${{\dot{\text{V}}}}$O_2_ plateau in the ramp test, as shown by our results.

### Influence of recovery phase duration

Prior high-intensity exercise above the lactate threshold has been repeatedly shown to accelerate overall ${{\dot{\text{V}}}}$O_2_ kinetics and reduce the accumulation of blood lactate concentration during a subsequent exercise bout (Bailey et al. 2009; Burnley et al. 2005, 2011). The underlying mechanism of this exercise-induced effect is the subject of intense debate and currently obscure (Bailey et al. 2009). As Bailey et al. (2009) and Burnley et al. (2005) emphasized, myriad physiological changes, including among others enhanced blood flow and muscle O_2_ availability, increased activity of oxidative muscle enzymes, and altered recruitment profiles of motor units may be underlying mechanism. A significant increase in time to the limit of exercise tolerance (Jones et al. 2003) and mean power output (Burnley et al. 2005) during an exercise bout following prior high-intensity exercise was observed in regularly active individuals and well-trained cyclists, respectively. Participants in both studies exhibited mild elevation in blood lactate concentration of approximately 2.5–3 mmol·L^−1^ at the onset of the subsequent exercise bout (Burnley et al. 2005; Jones et al. 2003). In contrast, Koppo and Bouckaert (2002) as well as Wilkerson et al. (2004) indicated that prior exercise producing a blood lactate concentration of approximately 6–7 mmol·L^−1^ had no effect respectively a negative effect on the time to the limit of exercise tolerance during the subsequent exercise bout. Thus, an appropriate combination of prior exercise intensity and recovery phase duration is of paramount importance, and severe lactic acidosis at the onset of the subsequent exercise bout may be accompanied by unchanged or reduced physical performance.

By implementing a recovery phase lasting until blood lactate concentration was ≤ 4 mmol·L^−1^, the present study aimed to establish an optimal condition for faster ${{\dot{\text{V}}}}$O_2_ kinetics during the verification phase and to increase the probability of a successful ${{\dot{\text{V}}}}$O_2max_ verification. However, considering the results of the present study, little added value could be attributed to both the sub-peak and supra-peak verification phases for the determination of 'true' ${{\dot{\text{V}}}}$O_2max_. Thus, overall ${{\dot{\text{V}}}}$O_2peak_ achieved in both verification phases did not show any significant increase compared to ${{\dot{\text{V}}}}$O_2peak_ from the ramp test. Considering the time to the limit of exercise tolerance of the supra-peak verification phase, compared to the study by Wagner et al. (2021) using an identical verification intensity but a remarkably shorter recovery phase duration, our participants sustained the verification phase for approximately 30 s longer. This may be related to an improved recovery, as our participants showed substantially lower blood lactate concentrations, heart rate, and rate of perceived exertion prior to the verification phase. However, despite individual adjustment of the recovery phase duration based on physiological exhaustion markers, the supra-peak verification phase in our study was preliminary terminated, i.e., a duration of at least 2:00 min was not reached. In conclusion, despite individually timed recovery phase duration, the used verification phase protocol adds little value to the determination of 'true' ${{\dot{\text{V}}}}$O_2max_. The benefit of a lactate-dependent recovery phase duration is therefore debatable, as it reduces the practicability in clinical routine due to increased time expenditure and amount of required blood samples.

### Generalizability of the results

It is crucial to emphasize that the present study, as well as the referenced studies supporting our findings, focused mainly on healthy adults. Consequently, the generalizability of the finding that both a sub-peak and a supra-peak verification phase do not contribute substantially to determining 'true' ${{\dot{\text{V}}}}$O_2max_ is limited to trained individuals without underlying health conditions. In the context of clinical populations, studies have investigated the usefulness of a verification phase in obese adults (Sawyer et al. 2015), patients with chronic heart disease (Bowen et al. 2012), cancer survivors (Schneider et al. 2020), as well as children, adolescents, and adults with cystic fibrosis (Saynor et al. 2013; Causer et al. 2018). While mean ${{\dot{\text{V}}}}$O_2peak_ values achieved during the ramp test and verification phase were mostly not significantly different, on an individual level, the verification phase elicited a higher ${{\dot{\text{V}}}}$O_2peak_ values compared to the previous ramp test in 20–66% of the participants (Saynor et al. 2013; Bowen et al. 2012; Sawyer et al. 2015; Schneider et al. 2020). Thus, assuming an underestimation of ${{\dot{\text{V}}}}$O_2peak_ during a ramp test in clinical populations, the use of a verification phase may be more justified. In clinical populations a verification phase could serve two purposes. First, it could be used to determine 'true' ${{\dot{\text{V}}}}$O_2max_. It is crucial to emphasize that a higher ${{\dot{\text{V}}}}$O_2_ value measured during the verification phase compared to the ramp test neither confirms the accuracy of the ${{\dot{\text{V}}}}$O_2peak_ achieved during the ramp test nor provides a definitive ${{\dot{\text{V}}}}$O_2max_ value. For example, probability that the participant may not have reached the ${{\dot{\text{V}}}}$O_2peak_ during the verification phase due to slow ${{\dot{\text{V}}}}$O_2_ kinetics is still substantial (Caputo and Denadai 2008). The second purpose of using a verification phase in a clinical population could be to increase the chances of detecting falsely measured ${{\dot{\text{V}}}}$O_2peak_ during the incremental protocol. Risk stratification and risk prediction models are all based on ${{\dot{\text{V}}}}$O_2peak_ derived from incremental testing, rather than ${{\dot{\text{V}}}}$O_2max_. However, some guidelines suggest certain thresholds to guide clinical decision making (Mancini et al. 1991). Consequently, in some patients, a verification phase test could be beneficial to support clinical decisions for the patient. However, it should be noted that clinical decisions, e.g., for heart transplantation, are not based on a single value but on the whole clinical picture.

### Strengths and limitations

In addition to examining both sexes, strengths of the present study included investigating the benefit of a sub-peak verification phase in addition to a supra-peak verification phase, implementing an exhaustion-dependent recovery duration, analyzing the data collected at the individual level, and considering the added value of a verification phase beyond a ${{\dot{\text{V}}}}$O_2_ plateau during the ramp test.

The inclusion of only well-trained participants in the present study has to be acknowledged as a noteworthy limitation. Our findings can thus not be generalized to exercise naïve individuals, clinical populations and older adults unaccustomed to reaching the limits of their exercise tolerance.

## Conclusion

In well-trained male and female adults, conducting a sub-peak verification phase following a ramp test may add little value to determining 'true' ${{\dot{\text{V}}}}$O_2max_, while a supra-peak verification phase may add no value. Despite the use of a verification protocol in which factors of intensity and recovery phase duration were implemented optimally and an individual-level analysis was performed, no clear additional benefit was seen by performing a verification phase. Conclusively, the little added value of conducting a sub-peak verification phase for determining ${{\dot{\text{V}}}}$O_2max_ barely justifies the enhanced physical strain, time, and financial effort. Further, conducting a supra-peak verification phase showed potential value in only one of the 31 tests performed. Here, confirmation of ${{\dot{\text{V}}}}$O_2max_ would already have been possible by secondary ${{\dot{\text{V}}}}$O_2max_ criteria. Since a verification phase inflicts substantial additional burden on all participants in addition to the use of ${{\dot{\text{V}}}}$O_2_ plateau and secondary ${{\dot{\text{V}}}}$O_2max_ criteria, its use might also be questionable. We infer that performing a verification phase in well-trained and highly motivated adults to determine 'true' ${{\dot{\text{V}}}}$O_2max_ can be omitted.

## Data Availability

Data will be provided upon reasonable request.

## Abbreviations

CPET: Cardiopulmonary exercise testing
PPO: Peak power output
${{\dot{\text{V}}}}$O_2max_: Maximum oxygen uptake
${{\dot{\text{V}}}}$O_2peak_: Highest oxygen uptake
SD: Standard deviation
